# Supplementary material for: Effectiveness of Internet- and Mobile-Based Cognitive Behavioral Therapy to Reduce Suicidal Ideation and Behaviors: Protocol for a Systematic Review and Meta-Analysis of Individual Participant Data
Source: Int J Environ Res Public Health. 2020 Jul 17;17(14):5179. doi: 10.3390/ijerph17145179 (PMC7399870; doi:10.3390/ijerph17145179)
Supplement: Supplementary file 1 [file ijerph-17-05179-s001.pdf]

Supplementary Materials

Table S1. Search strings for CENTRAL, PsycINFO, Embase and Pubmed.

	**CENTRAL**		**PsycINFO via Ebsco**		**Embase via Ovid**
#1	MeSH descriptor: [Computers] explode all trees	S1	MA computers	1	computer.sh.
#2	MeSH descriptor: [Software] explode all trees	S2	MA software	2	software.sh.
#3	MeSH descriptor: [Internet] explode all trees	S3	MA internet	3	internet.sh.
#4	MeSH descriptor: [Web Browser] explode all trees	S4	MA web browser	4	web browser.sh.
#5	MeSH descriptor: [Technology] explode all trees	S5	MA technology	5	technology.sh.
#6	MeSH descriptor: [Cell Phone] explode all trees	S6	MA cell phone	6	mobile phone.sh.
#7	MeSH descriptor: [Mobile Applications] explode all trees	S7	MA mobile applications	7	mobile application.sh.
#8	MeSH descriptor: [Therapy, Computer-Assisted] explode all trees	S8	MA therapy, computer-assisted	8	computer-assisted therapy.sh.
#9	MeSH descriptor: [Telemedicine] explode all trees	S9	MA telemedicine	9	telemedicine.sh.
#10	MeSH descriptor: [Medical Informatics] explode all trees	S10	MA telerehabilitation	10	telerehabilitation.sh.
#11	MeSH descriptor: [Distance Counseling] explode all trees	S11	MA medical informatics	11	medical informatics.sh.
#12	(technolog*):ti,ab,kw	S12	MA distance counseling	12	e-counseling.sh.
#13	(software):ti,ab,kw	S13	TI technolog* OR AB technolog*	13	technolog*.ti. or technolog*.ab.
#14	(web):ti,ab,kw	S14	TI software OR AB software	14	software.ti. or software.ab.
#15	("app-based" OR "app based"):ti,ab,kw	S15	TI web OR AB web	15	web.ti. or web.ab.
#16	(internet):ti,ab,kw	S16	TI "app-based" OR AB "app-based" OR TI "app based" OR AB "app based"	16	("app-based" or "app based").ti. or ("app-based" or "app based") .ab.
#17	(online):ti,ab,kw	S17	TI internet OR AB internet	17	internet.ti. or internet.ab.
#18	(computer*):ti,ab,kw	S18	TI online OR AB online	18	online.ti. or online.ab.
#19	(cyber):ti,ab,kw	S19	TI computer* OR AB computer*	19	computer*.ti. or computer*.ab.
#20	(electronic):ti,ab,kw	S20	TI cyber OR AB cyber	20	cyber.ti. or cyber.ab.
#21	("world wide web" OR www):ti,ab,kw	S21	TI electronic OR AB electronic	21	electronic.ti. or electronic.ab.
#22	(net):ti,ab,kw	S22	TI "world wide web" OR AB "world wide web" OR TI www OR AB www	22	("world wide web" or www).ti. or ("world wide web" or www).ab.
#23	(digital):ti,ab,kw	S23	TI net OR AB net	23	net.ti. or net.ab.
#24	(virtual):ti,ab,kw	S24	TI digital OR AB digital	24	digital.ti. or digital.ab.
#25	(website):ti,ab,kw	S25	TI virtual OR AB virtual	25	virtual.ti. or virtual.ab.
#26	(chat):ti,ab,kw	S26	TI website OR AB website	26	website.ti. or website.ab.
#27	(forum):ti,ab,kw	S27	TI chat OR AB chat	27	chat.ti. or chat.ab.
#28	(E-Mail OR email):ti,ab,kw	S28	TI forum OR AB forum	28	forum.ti. or forum.ab.
#29	(SMS):ti,ab,kw	S29	TI e-mail OR AB e-mail OR TI email OR AB email	29	(e-mail or email).ti. or (e-mail or email).ab.
#30	("text messag*" OR textmessag*):ti,ab,kw	S30	TI SMS OR AB SMS	30	SMS.ti. or SMS.ab.
#31	(mobile):ti,ab,kw	S31	TI "text messag*" OR AB	31	(text messag*" or

			"text messag*" OR TI textmessag* OR AB textmessag*		textmessag*).ti. or (text messag*" or textmessag*).ab.
#32	(smartphone):ti,ab,kw	S32	TI mobile OR AB mobile	32	mobile.ti. or mobile.ab.
#33	(phone):ti,ab,kw	S33	TI smartphone OR AB smartphone	33	smartphone.ti. or smartphone.ab.
#34	(e-therap*):ti,ab,kw	S34	TI phone OR AB phone	34	phone.ti. or phone.ab.
#35	("e-mental health" OR "emental health"):ti,ab,kw	S35	TI e-therap* OR AB e-therap*	35	e-therap*.ti. or e-therap*.ab.
#36	(e-health OR ehealth):ti,ab,kw	S36	TI "e-mental health" OR AB "e-mental health" OR TI "emental health" OR AB "emental health"	36	("e-mental health" or "emental health").ti. or ("e-mental health" or "emental health").ab.
#37	(mhealth OR m-health):ti,ab,kw	S37	TI e-health OR AB e-health OR TI ehealth OR AB ehealth	37	(e-health or ehealth).ti. or (e-health or ehealth).ab.
#38	(tele*):ti,ab,kw	S38	TI mhealth OR AB mhealth OR TI m-health OR AB m-health	38	(mhealth or m-health).ti. or (mhealth or m-health).ab.
#39	(iCBT OR i-CBT):ti,ab,kw	S39	TI tele* OR AB tele*	39	tele*.ti. or tele*.ab.
#40	C(CBT OR c-CBT):ti,ab,kw	S40	TI iCBT OR AB iCBT OR TI i-CBT OR AB i-CBT	40	(iCBT or i-CBT).ti. or (iCBT or i-CBT).ab.
#41	("personal digital assist*" OR PDA):ti,ab,kw	S41	TI cCBT OR AB cCBT OR TI c-CBT OR AB c-CBT	41	(cCBT or c-CBT).ti. or (cCBT or c-CBT).ab.
#42	("cell* phone*"):ti,ab,kw	S42	TI "personal digital assist*" OR AB "personal digital assist*" OR TI PDA OR AB PDA	42	("personal digital assist*" or PDA).ti. or ("personal digital assist*" or PDA).ab.
		S43	TI "cell* phone*" OR AB "cell* phone*"	43	("cell phone" or "cell phones" or "cellular phone" or "cellular phones").ti. or ("cell phone" or "cell phones" or "cellular phone" or "cellular phones").ab.
		S44	SU computer applications	44	psychological software.sh.
		S45	SU internet	45	internet.sh.
		S46	SU information and communication technology	46	information technology.sh.
		S47	SU mobile applications	47	mobile health application.sh.
		S48	SU mobile devices		
		S49	SU mobile health		
		S50	SU mobile phones		
		S51	SU mobile technology		
		S52	SU telemedicine		
		S53	SU online therapy		
		S54	SU computer assisted therapy		
#43	#1 OR #2 OR #3 OR #4 OR #5 OR #6 OR #7 OR #8 OR #9 OR #10 OR #11 OR #12 OR #13 OR #14 OR #15 OR #16 OR #17 OR #18 OR #19 OR #20 OR #21 OR #22 OR #23 OR #24 OR #25 OR #26 OR #27 OR #28 OR #29 OR #30 OR #31 OR #32 OR #33 OR #34 OR #35 OR #36 OR #37 OR #38 OR #39 OR #40 OR #41 OR #42	S55	S1 OR S2 OR S3 OR S4 OR S5 OR S6 OR S7 OR S8 OR S9 OR S10 OR S11 OR S12 OR S13 OR S14 OR S15 OR S16 OR S17 OR S18 OR S19 OR S20 OR S21 OR S22 OR S23 OR S24 OR S25 OR S26 OR S27 OR S28 OR S29 OR S30 OR S31 OR S32 OR S33 OR S34 OR S35 OR S36 OR S37 OR S38 OR S39 OR S40 OR S41 OR S42 OR S43 OR S44 OR S45 OR S46 OR S47 OR S48 OR S49 OR S50 OR S51 OR S52 OR S53 OR S54	48	1 or 2 or 3 or 4 or 5 or 6 or 7 or 8 or 9 or 10 or 11 or 12 or 13 or 14 or 15 or 16 or 17 or 18 or 19 or 20 or 21 or 22 or 23 or 24 or 25 or 26 or 27 or 28 or 29 or 30 or 31 or 32 or 33 or 34 or 35 or 36 or 37 or 38 or 39 or 40 or 41 or 42 or 43 or 44 or 45 or 46 or 47
#44	MeSH descriptor: [Suicide] explode all trees	S56	MA suicide	49	suicide.sh.
#45	MeSH descriptor: [Self-	S57	MA self-injurious behavior	50	self-injurious behavior.sh.

	Injurious Behavior] explode all trees				
#46	MeSH descriptor: [Suicidal Ideation] explode all trees	S58	MA suicidal ideation	51	suicidal ideation.sh.
#47	MeSH descriptor: [Suicide, Attempted] explode all trees	S59	MA suicide, attempted	52	suicide attempt.sh.
#48	(suicid*):ti,ab,kw	S60	TI suicid* OR AB suicid*	53	suicid*.ti. or suicid*.ab.
#49	(self-injur* OR selfinjur*):ti,ab,kw	S61	TI self-injur* OR AB self-injur* OR TI selfinjur* OR AB selfinjur*	54	(self-injur* or selfinjur*).ti. or (self-injur* or selfinjur*).ab.
#50	(self-harm OR selfharm):ti,ab,kw	S62	TI self-harm OR AB self-harm OR TI selfharm OR AB selfharm	55	(self-harm or selfharm).ti. or (self-harm or selfharm).ab.
#51	(self-mutilation OR selfmutilation):ti,ab,kw	S63	TI self-mutilation OR AB self-mutilation OR TI selfmutilation OR AB selfmutilation	56	(self-mutilation or selfmutilation).ti. or (self-mutilation or selfmutilation).ab.
#52	(auto-mutilation OR automutilation):ti,ab,kw	S64	TI auto-mutilation OR AB auto-mutilation OR TI automutilation OR AB automutilation	57	(auto-mutilation or automutilation).ti. or (auto-mutilation or automutilation).ab.
		S65	SU self-mutilation	58	automutilation.sh.
		S66	SU suicide		
		S67	SU suicidal ideation		
		S68	SU attempted suicide		
		S69	SU suicidology		
		S70	SU self-injurious behavior		
		S71	SU suicide prevention		
#53	#44 OR #45 OR #46 OR #47 OR #48 OR #49 OR #50 OR #51 OR #52	S72	S56 OR S57 OR S58 OR S59 OR S60 OR S61 OR S62 OR S63 OR S64 OR S65 OR S66 OR S67 OR S68 OR S69 OR S70 OR S71	59	49 or 50 or 51 or 52 or 53 or 54 or 55 or 56 or 57 or 58
#54	MeSH descriptor: [Randomized Controlled Trials as Topic] explode all trees	S73	MA randomized controlled trials as topic	60	randomized controlled trial.sh.
#55	MeSH descriptor: [Clinical Trials as Topic] explode all trees	S74	MA clinical trials as topic	61	clinical trial.sh.
#56	("randomized controlled trial"):pt	S75	TI RCT OR AB RCT	62	RCT.ti. or RCT.ab.
#57	("controlled clinical trial"):pt	S76	TI random* OR AB random*	63	random*.ti. or random*.ab.
#58	(clinical trial):pt	S77	TI trial OR AB trial	64	trial.ti. or trial.ab.
#59	(clinical trial protocol):pt				
#60	(clinical study):pt				
#61	(random allocation):pt				
#62	(RCT):ti,ab,kw				
#63	(clinical trial):ti,ab,kw				
#64	(random*):ti,ab,kw				
#65	("trial"):ti,ab,kw				
#66	#54 OR #55 OR #57 OR #58 OR #59 OR #60 OR #61 OR #62 OR #63 OR #64 OR #65	S78	S73 OR S74 OR S75 OR S76 OR S77	65	60 OR 61 OR 62 OR 63 OR 64
#67	#43 AND #53 AND #66	S79	S55 AND S72 AND S78	66	48 AND 59 AND 65

Note. MH/MA: MeSH Term; TI/ti: title; AB/ab: abstract; SU: subject terms; sh: subject headiings; PT/pt: publication type; ti,ab,kw: title, abstract, keywords.

Pubmed:

(computers[MeSH Terms] OR software[MeSH Terms] OR internet[MeSH Terms] OR web browser[MeSH Terms] OR technology[MeSH Terms] OR cell phone[MeSH Terms] OR mobile applications[MeSH Terms] OR therapy, computer-assisted[MeSH Terms] OR telemedicine[MeSH Terms] OR telerehabiliation[MeSH Terms] OR medical informatics[MeSH Terms] OR distance counseling[MeSH Terms] OR technolog*[Title/Abstract] OR software[Title/Abstract] OR web[Title/Abstract] OR “app-based”[Title/Abstract] OR “app based”[Title/Abstract]

OR internet[Title/Abstract] OR online[Title/Abstract] OR computer*[Title/Abstract] OR cyber[Title/Abstract] OR electronic[Title/Abstract] OR "world wide web"[Title/Abstract] OR www[Title/Abstract] OR net[Title/Abstract] OR digital[Title/Abstract] OR virtual[Title/Abstract] OR website[Title/Abstract] OR chat[Title/Abstract] OR forum[Title/Abstract] OR e-mail[Title/Abstract] OR email[Title/Abstract] OR SMS[Title/Abstract] OR "text messag*"[Title/Abstract] OR textmessag*[Title/Abstract] OR mobile[Title/Abstract] OR smartphone[Title/Abstract] OR phone[Title/Abstract] OR e-therap*[Title/Abstract] OR "e-mental health"[Title/Abstract] OR "emental health"[Title/Abstract] OR e-health[Title/Abstract] OR ehealth[Title/Abstract] OR mhealth[Title/Abstract] OR m-health[Title/Abstract] OR tele-care[Title/Abstract] OR telecare[Title/Abstract] OR tele-health[Title/Abstract] OR telehealth[Title/Abstract] OR tele-medicine[Title/Abstract] OR telemedicine[Title/Abstract] OR tele-rehabilitation[Title/Abstract] OR telerehabilitation[Title/Abstract] OR telephone [Title/Abstract] OR iCBT[Title/Abstract] OR i-CBT[Title/Abstract] OR cCBT[Title/Abstract] OR c-CBT[Title/Abstract] OR "personal digital assist*"[Title/Abstract] OR PDA[Title/Abstract] OR "cell* phone*"[Title/Abstract]) AND (suicide[MeSH Terms] OR "self-injurious behavior"[MeSH Terms] OR "suicidal ideation"[MeSH Terms] OR "suicide, attempted"[MeSH Terms] OR suicid*[Title/Abstract] OR self-injur*[Title/Abstract] OR selfinjur*[Title/Abstract] OR self-harm[Title/Abstract] OR selfharm[Title/Abstract] OR self-mutilation[Title/Abstract] OR selfmutilation[Title/Abstract] OR auto-mutilation[Title/Abstract] OR automutilation[Title/Abstract]) AND ("randomized controlled trials as topic"[MeSH Terms] OR "clinical trials as topic"[MeSH Terms] OR "randomized controlled trial"[Publication Type] OR "controlled clinical trial"[Publication Type] OR "clinical trial"[Publication Type] OR "clinical trial protocol"[Publication Type] OR "clinical study"[Publication Type] OR RCT[Title/Abstract] OR random*[Title/Abstract] OR trial [Title/Abstract])

© 2020 by the authors. Submitted for possible open access publication under the terms and conditions of the Creative Commons Attribution (CC BY) license (http://creativecommons.org/licenses/by/4.0/).
